# Supplementary material for: Microglial Senescence and Activation in Healthy Aging and Alzheimer’s Disease: Systematic Review and Neuropathological Scoring
Source: Cells. 2023 Dec 12;12(24):2824. doi: 10.3390/cells12242824 (PMC10742050; doi:10.3390/cells12242824)
Supplement: Supplementary file 1 [file cells-12-02824-s001.zip › cells-2721675-supplementary.pdf]

Table S1c-d. NIH quality assessment tool for Systematic Reviews and Meta-Analyses

Criteria	Is the review based on a focused question that is adequately formulated and described (e.g., PICO format)?	Were eligibility criteria for included and excluded studies predefined and specified?	Did the literature search strategy use a comprehensive, systematic approach?	Were titles, abstracts, and full-text articles dually and independently reviewed for inclusion and exclusion to minimize bias?	Was the quality of each included study rated independently by two or more reviewers using a standard method to appraise its internal validity?	Were the included studies listed along with important characteristics and results of each study?	Was publication bias assessed?
	**Yes**	**Yes**	**Yes**	**Yes**	**Yes**	**Yes**	**Yes**

Quality Rating (Good, Fair, or Poor)	Rater #1 A.M.	Rater #2 A.G.	Rater #3 T.E.P.
	**Good**	**Good**	**Good**

Table S1e. NIH quality assessment tool for Case-Control Studies

(A) No = no provided information; Partially = some information is provided; Yes = presence of objective inclusion and exclusion criteria
(B) No = no provided information; Partially = information is provided either incomplete or without complete data; Yes = presence of clinical and neuroimaging finding, cognitive assessment, follow-up information that were similar to the cases.

Autor	Study type	**Was the research question or objective in this paper clearly stated and appropriate?**	**Was the study population clearly specified and defined? (A)**	**Did the authors include a sample size justification?**	**Were controls selected or recruited from the same or similar population that gave rise to the cases (including the same timeframe)? (B)**	**Were the definitions, inclusion and exclusion criteria, algorithms or processes used to identify or select cases and controls valid, reliable, and implemented consistently across all study participants? (A)**	**Were the cases clearly defined and differentiated from controls? (A)**	**If less than 100 percent of eligible cases and/or controls were selected for the study, were the cases and/or controls randomly selected from those eligible?**	**Was there use of concurrent controls?**	**Quality Rating (Good, Fair, or Poor)**
**Aghaizu et al. (2023)**	Case-Control	Yes	Yes	%	Yes	Yes	Yes	%	Yes	Rater #1 A.M.: Good<br>Rater #2 A.G.: Good
**Pascoal et al. (2021)**	Case-Control	Yes	Yes	%	Yes	Yes	Yes	%	Yes	Rater #1 A.M.: Good<br>Rater #2 A.G.: Good

**March-Diaz et al. (2021)**	Case-Control	Yes	Partially	%	Partially	Partially	Yes	%	Yes	Rater #1 A.M.: Good<br>Rater #2 A.G.: Good
**Dhawan et al. (2012)**	Case-Control	Yes	Partially	%	Partially	Partially	Yes	%	Yes	Rater #1 A.M.: Good<br>Rater #2 A.G.: Good
**Bachstetter et al. (2017)**	Case-Control	Yes	Yes	%	Yes	Yes	Yes	%	Yes	Rater #1 A.M.: Good<br>Rater #2 A.G.: Good
**Zeineh et al. (2015)**	Case-Control	Yes	Yes	%	Yes	Yes	Yes	%	Yes	Rater #1 A.M.: Good<br>Rater #2 A.G.: Good
**Barroeta-Espar et al. (2019)**	Case-Control	Yes	Yes	%	Yes	Yes	Yes	%	Yes	Rater #1 A.M.: Good<br>Rater #2 A.G.: Good
**Raj et al. (2017)**	Case-Control	Yes	Yes	%	Yes	Yes	Yes	%	Yes	Rater #1 A.M.: Good<br>Rater #2 A.G.: Good
**Mukherhjee et al. (2019)**	Case-Control	Yes	Partially	%	Partially	Partially	Partially	%	Yes	Rater #1 A.M.: Good<br>Rater #2 A.G.: Good
**Molina-Martinez et al. (2020)**	Case-Control	Yes	Yes	%	Yes	Yes	Yes	%	Yes	Rater #1 A.M.: Good<br>Rater #2 A.G.: Good
**Jiang et al. (2022)**	Case-Control	Yes	Yes	%	Yes	Yes	Yes	%	Yes	Rater #1 A.M.: Good<br>Rater #2 A.G.: Good
**Sheng et al. (2017)**	Case-Control	Yes	Yes	%	Yes	Yes	Yes	%	Yes	Rater #1 A.M.: Good<br>Rater #2 A.G.: Good
**Parhizkar et al. (2019)**	Case-Control	Yes	Yes	%	Yes	Yes	Yes	%	No	Rater #1 A.M.: Good<br>Rater #2 A.G.: Good
**Sims et al. (2017)**	Case-Control	Yes	Partially	%	No	Yes	Yes	%	Yes	Rater #1 A.M.: Good<br>Rater #2 A.G.: Good
**Whitaker Cohn et al. (2021)**	Case-Control	Yes	Yes	%	No	Yes	Yes	%	No	Rater #1 A.M.: Good<br>Rater #2 A.G.: Good
**Srinivasan et al. (2020)**	Case-Control	Yes	Yes	%	Yes	Yes	Yes	%	Yes	Rater #1 A.M.: Good<br>Rater #2 A.G.: Good
**Neumann et al. (2022)**	Case-Control	Yes	Yes	%	No	Yes	No	%	Yes	Rater #1 A.M.: Good<br>Rater #2 A.G.: Good
**Walker et al. (2001)**	Case-Control	Yes	Yes	%	No	Yes	No	%	Yes	Rater #1 A.M.: Good<br>Rater #2 A.G.: Good
**Shahidehpour et al. (2021)**	Case-Control	Yes	Yes	%	Yes	Yes	Yes	%	Yes	Rater #1 A.M.: Good<br>Rater #2 A.G.: Good
**Hu et al. (2021)**	Case-Control	Yes	Yes	%	Yes	Yes	Yes	%	Yes	Rater #1 A.M.: Good<br>Rater #2 A.G.: Good

**Smith et al. (2013)**	Case-Control	Yes	Yes	%	Partially	Partially	Partially	%	Partially	Rater #1 A.M.: Good Rater #2 A.G.: Good
**Lopes et al. (2008)**	Case-Control	Yes	Yes	%	Yes	Yes	Yes	%	Yes	Rater #1 A.M.: Good Rater #2 A.G.: Good
**Walker et al. (2020)**	Case-Control	Yes	Yes	%	Yes	Yes	Yes	%	Yes	Rater #1 A.M.: Good Rater #2 A.G.: Good
**Streit et al. (2009)**	Case-Control	Yes	Yes	%	Yes	Yes	Yes	%	Yes	Rater #1 A.M.: Good Rater #2 A.G.: Good
**Flanary et al. (2007)**	Case-Control	Yes	Yes	%	Yes	Yes	Yes	%	Yes	Rater #1 A.M.: Good Rater #2 A.G.: Good
**Felsky et al. (2019)**	Case-Control	Yes	Yes	%	Yes	Yes	Partially	%	No	Rater #1 A.M.: Good Rater #2 A.G.: Good
**Bonham et al. (2019)**	Case-Control	Yes	Yes	%	Yes	Yes	Yes	%	Yes	Rater #1 A.M.: Good Rater #2 A.G.: Good
**Li et al. (2020)**	Case-Control	Yes	Yes	%	Yes	Yes	Yes	%	Yes	Rater #1 A.M.: Good Rater #2 A.G.: Good
**Griciuc et al. (2013)**	Case-Control	Yes	Yes	%	Yes	Yes	Yes	%	Yes	Rater #1 A.M.: Good Rater #2 A.G.: Good
**Xie et al. (2021)**	Case-Control	Yes	Yes	%	Yes	Yes	Yes	%	Yes	Rater #1 A.M.: Good Rater #2 A.G.: Good
**Olah et al. (2020)**	Case-Control	Yes	Yes	%	Yes	Yes	Partially	%	Partially	Rater #1 A.M.: Good Rater #2 A.G.: Good
**Tischer et al. (2016)**	Case-Control	Yes	Yes	%	Yes	Yes	Yes	%	Yes	Rater #1 A.M.: Good Rater #2 A.G.: Good
**An et al. (2022)**	Case-Control	Yes	Partially	%	Partially	Partially	Partially	%	Partially	Rater #1 A.M.: Good Rater #2 A.G.: Good
**Davies et al. (2017)**	Case-Control	Yes	Yes	%	Yes	Yes	Yes	%	Yes	Rater #1 A.M.: Good Rater #2 A.G.: Good
**Munoz-Castro et al. (2022)**	Case-Control	Yes	Yes	%	Yes	Yes	Yes	%	Yes	Rater #1 A.M.: Good Rater #2 A.G.: Good
**Kloske et al. (2021)**	Case-Control	Yes	Yes	%	Yes	Yes	Yes	%	Yes	Rater #1 A.M.: Good Rater #2 A.G.: Good
**Bachstetter et al. (2015)**	Case-Control	Yes	Yes	%	Yes	Yes	Yes	%	Yes	Rater #1 A.M.: Good Rater #2 A.G.: Good
**Kaneshwaran et al. (2019)**	Case-Control	Yes	Yes	%	Yes	Yes	Yes	%	Yes	Rater #1 A.M.: Good Rater #2 A.G.: Good

**Fadul et al. (2020)**	Case-Control	Yes	Yes	%	Partially	Partially	Partially	%	Yes	Rater #1 A.M.: Good<br>Rater #2 A.G.: Good
**DiPatre et al. (1997)**	Case-Control	Yes	Yes	%	Yes	Yes	Yes	%	Yes	Rater #1 A.M.: Good<br>Rater #2 A.G.: Good
**Hendrickx et al. (2017)**	Case-Control	Yes	Yes	%	Yes	Yes	Yes	%	Yes	Rater #1 A.M.: Good<br>Rater #2 A.G.: Good
**Krasemann et al. (2017)**	Case-Control	Yes	Yes	%	Yes	Yes	Yes	%	Yes	Rater #1 A.M.: Good<br>Rater #2 A.G.: Good
**Satoh et al. (2016)**	Case-Control	Yes	Yes	%	Yes	Yes	Yes	%	Partial ly	Rater #1 A.M.: Good<br>Rater #2 A.G.: Good
**Marschallinger et al. (2020)**	Case-Control	Yes	Yes	%	Yes	Yes	Partially	%	Yes	Rater #1 A.M.: Good<br>Rater #2 A.G.: Good
**Olah et al. (2018)**	Case-Control	Yes	Yes	%	Yes	Yes	Yes	%	Yes	Rater #1 A.M.: Good<br>Rater #2 A.G.: Good

Supplementary Table. S2. Pubmed and Scopus databases search strings with MeSH terms and classical terms.

**PubMed**	(((("microglial"[All Fields] OR "microglials"[All Fields]) AND ("aging"[MeSH Terms] OR "aging"[All Fields] OR "senescence"[All Fields] OR "senesce"[All Fields] OR "senesced"[All Fields] OR "senescences"[All Fields] OR "senescent"[All Fields] OR "senescents"[All Fields] OR "senesces"[All Fields] OR "senescing"[All Fields])) OR (("microglial"[All Fields] OR "microglials"[All Fields]) AND ("activable"[All Fields] OR "activate"[All Fields] OR "activated"[All Fields] OR "activates"[All Fields] OR "activating"[All Fields] OR "activation"[All Fields] OR "activations"[All Fields] OR "activator"[All Fields] OR "activator s"[All Fields] OR "activators"[All Fields] OR "active"[All Fields] OR "actived"[All Fields] OR "actively"[All Fields] OR "actives"[All Fields] OR "activities"[All Fields] OR "activity s"[All Fields] OR "activitys"[All Fields] OR "motor activity"[MeSH Terms] OR ("motor"[All Fields] AND "activity"[All Fields]) OR "motor activity"[All Fields] OR "activity"[All Fields]))) AND ("human s"[All Fields] OR "humans"[MeSH Terms] OR "humans"[All Fields] OR "human"[All Fields] OR ("human s"[All Fields] OR "humans"[MeSH Terms] OR "humans"[All Fields] OR "human"[All Fields])) AND ("alzheimer disease"[MeSH Terms] OR ("alzheimer"[All Fields] AND "disease"[All Fields]) OR "alzheimer disease"[All Fields] OR ("alzheimer disease"[MeSH Terms] OR ("alzheimer"[All Fields] AND "disease"[All Fields]) OR "alzheimer disease"[All Fields] OR ("alzheimer s"[All Fields] AND "disease"[All Fields]) OR "alzheimer s disease"[All Fields]) OR ("healthy aging"[MeSH Terms] OR ("healthy"[All Fields] AND "aging"[All Fields]) OR "healthy aging"[All Fields]) OR (("healthies"[All Fields] OR "healthy"[All Fields]) AND ("controling"[All Fields] OR "controllability"[All Fields] OR "controllable"[All Fields] OR "controllably"[All Fields] OR "controller"[All Fields] OR "controller s"[All Fields] OR "controllers"[All Fields] OR "controlling"[All Fields] OR "controls"[All Fields] OR "prevention

	and control"[MeSH Subheading] OR ("prevention"[All Fields] AND "control"[All Fields]) OR "prevention and control"[All Fields] OR "control"[All Fields] OR "control groups"[MeSH Terms] OR ("control"[All Fields] AND "groups"[All Fields]) OR "control groups"[All Fields])))) AND ((humans[Filter]) AND (english[Filter]))
**Scopus**	TITLE-ABS-KEY ("microglial senescence" OR "microglial activation") AND TITLE-ABS-KEY ("Human") AND TITLE-ABS-KEY ("Alzheimer disease" OR "Alzheimer's disease" OR "healthy aging" OR "Healthy Controls") AND (LIMIT-TO (EXACTKEYWORD , "Human")) AND (LIMIT-TO (LANGUAGE , "English"))

Supplementary Table. S3 PRISMA Checklist 2020. *From* Page MJ, McKenzie JE, Bossuyt PM, Boutron I, Hoffmann TC, Mulrow CD, et al. The PRISMA 2020 statement: an updated guideline for reporting systematic reviews. *BMJ* 2021;372:n71. doi: 10.1136/bmj.n71

Section and Topic	Item #	Checklist item	Pages or section where item is reported
**TITLE**			
Title	1	Identify the report as a systematic review.	1
**ABSTRACT**			
Abstract	2	See the PRISMA 2020 for Abstracts checklist.	1
**INTRODUCTION**			
Rationale	3	Describe the rationale for the review in the context of existing knowledge.	1-3
Objectives	4	Provide an explicit statement of the objective(s) or question(s) the review addresses.	3
**METHODS**			
Eligibility criteria	5	Specify the inclusion and exclusion criteria for the review and how studies were grouped for the syntheses.	4-5-6
Information sources	6	Specify all databases, registers, websites, organisations, reference lists and other sources searched or consulted to identify studies. Specify the date when each source was last searched or consulted.	4-5-6
Search strategy	7	Present the full search strategies for all databases, registers and websites, including any filters and limits used.	4-5-6
Selection process	8	Specify the methods used to decide whether a study met the inclusion criteria of the review, including how many reviewers screened each record and each report retrieved, whether they worked independently, and if applicable, details of automation tools used in the process.	4-5-6
Data collection process	9	Specify the methods used to collect data from reports, including how many reviewers collected data from each report, whether they worked independently, any processes for obtaining or confirming data from study investigators, and if applicable, details of automation tools used in the process.	4-5-6
Data items	10a	List and define all outcomes for which data were sought. Specify whether all results that were compatible with each outcome domain in each study were sought (e.g. for all measures, time points, analyses), and if not, the methods used to decide which results to collect.	4-5-6
	10b	List and define all other variables for which data were sought (e.g. participant and intervention characteristics, funding sources). Describe any assumptions made about any missing or	4-5

Section and Topic	Item #	Checklist item	Pages or section where item is reported
		unclear information.	
Study risk of bias assessment	11	Specify the methods used to assess risk of bias in the included studies, including details of the tool(s) used, how many reviewers assessed each study and whether they worked independently, and if applicable, details of automation tools used in the process.	5-6
Effect measures	12	Specify for each outcome the effect measure(s) (e.g. risk ratio, mean difference) used in the synthesis or presentation of results.	NA
Synthesis methods	13a	Describe the processes used to decide which studies were eligible for each synthesis (e.g. tabulating the study intervention characteristics and comparing against the planned groups for each synthesis (item #5)).	4-5-6
	13b	Describe any methods required to prepare the data for presentation or synthesis, such as handling of missing summary statistics, or data conversions.	4-5-6
	13c	Describe any methods used to tabulate or visually display results of individual studies and syntheses.	NA
	13d	Describe any methods used to synthesize results and provide a rationale for the choice(s). If meta-analysis was performed, describe the model(s), method(s) to identify the presence and extent of statistical heterogeneity, and software package(s) used.	NA
	13e	Describe any methods used to explore possible causes of heterogeneity among study results (e.g. subgroup analysis, meta-regression).	NA
	13f	Describe any sensitivity analyses conducted to assess robustness of the synthesized results.	NA
Reporting bias assessment	14	Describe any methods used to assess risk of bias due to missing results in a synthesis (arising from reporting biases).	5
Certainty assessment	15	Describe any methods used to assess certainty (or confidence) in the body of evidence for an outcome.	4-5
**RESULTS**			
Study selection	16a	Describe the results of the search and selection process, from the number of records identified in the search to the number of studies included in the review, ideally using a flow diagram.	5
	16b	Cite studies that might appear to meet the inclusion criteria, but which were excluded, and explain why they were excluded.	5, Tables 1a and 1b
Study characteristics	17	Cite each included study and present its characteristics.	27-35
Risk of bias in studies	18	Present assessments of risk of bias for each included study.	Supplementary Materials
Results of individual studies	19	For all outcomes, present, for each study: (a) summary statistics for each group (where appropriate) and (b) an effect estimate and its precision (e.g. confidence/credible interval), ideally using structured tables or plots.	Table 1a and Table 1b Supplementary Materials
Results of syntheses	20a	For each synthesis, briefly summarise the characteristics and risk of bias among contributing studies.	Table 1a and Table 1b Supplementary Materials
	20b	Present results of all statistical syntheses conducted. If meta-analysis was done, present for each the summary estimate and its precision (e.g. confidence/credible interval) and measures of statistical heterogeneity. If comparing groups, describe the direction of the effect.	NA
	20c	Present results of all investigations of possible causes of heterogeneity among study results.	NA
	20d	Present results of all sensitivity analyses conducted to assess the robustness of the synthesized results.	NA
Reporting biases	21	Present assessments of risk of bias due to missing results (arising from reporting biases) for each synthesis assessed.	NA

Section and Topic	Item #	Checklist item	Pages or section where item is reported
Certainty of evidence	22	Present assessments of certainty (or confidence) in the body of evidence for each outcome assessed.	NA
**DISCUSSION**			
Discussion	23a	Provide a general interpretation of the results in the context of other evidence.	7-26
	23b	Discuss any limitations of the evidence included in the review.	25-26
	23c	Discuss any limitations of the review processes used.	5-6-7, 25-26
	23d	Discuss implications of the results for practice, policy, and future research.	25-26
**OTHER INFORMATION**			
Registration and protocol	24a	Provide registration information for the review, including register name and registration number, or state that the review was not registered.	INPLASY2023110064
	24b	Indicate where the review protocol can be accessed, or state that a protocol was not prepared.	DOI: 10.37766/inplasy2023.11.0064
	24c	Describe and explain any amendments to information provided at registration or in the protocol.	NA
Support	25	Describe sources of financial or non-financial support for the review, and the role of the funders or sponsors in the review.	39
Competing interests	26	Declare any competing interests of review authors.	39
Availability of data, code and other materials	27	Report which of the following are publicly available and where they can be found: template data collection forms; data extracted from included studies; data used for all analyses; analytic code; any other materials used in the review.	Dataset available at DOI: 10.5281/zenodo.10154403. data extracted from included studies 39
